# Factors and determinants associated with prevalence of stunting and thinness among adolescents of Tharparkar, Sindh, Pakistan: A community-based study

**DOI:** 10.1371/journal.pone.0318055

**Published:** 2025-04-23

**Authors:** Aisha Memon, Zulfiqar Ali Laghari, Ayaz Ali Samo

**Affiliations:** Department of Physiology, Faculty of Natural Sciences, University of Sindh, Jamshoro, Sindh, Pakistan; Dow University of Health Sciences, PAKISTAN

## Abstract

Stunting and thinness are significant public health concerns for developing countries, including Pakistan. Previously conducted studies in Tharparkar have focused on stunting, and thinness, in children, and micronutrient deficiency, However, there was a paucity of literature on prevalence rates and risk factors associated with stunting and thinness among adolescents in Tharparkar, Sindh, Pakistan. This study aimed to assess the demographic and dietary factors related to the prevalence of stunting and thinness among adolescents in Tharparkar. A community-based cross-sectional study was conducted from June 2022 to August 2022. Most populated Union councils of all sub-districts of Tharparkar district were selected for sampling. A multi-stage clustering sampling strategy was used. Healthy adolescents with ages≥10 to ≤19 years were included in the study. Anthropometry was performed using standard methods. Stunting and thinness were defined as per WHO criteria. Dietary assessments were conducted on a weekly recall basis. Mean Frequency and chi-square were computed using SPSS software. The overall mean age of the study participants was 14.11±2.43 years, and the mean weight was 36.842±8.83 kgs. The mean height of the study participants was 149.0151±11.27 centimeters. The mean height for age z score was -1.3094±1.17. The mean body mass index for age z score was -1.5473±1.27. Among 599 participants the overall prevalence of stunting and thinness was 26.7% and 35% respectively. Risk factors such as late adolescence (χ2=10.55 p=0.005), illiteracy, and less education (χ2=8.41 p=0.03), the rural area (χ2=3.92 p=0.04) significantly associated with an increased prevalence of stunting. The risk factors such as male (χ2=13.11 p<0.0001), infrequent consumption of eggs (χ2=5.2 p=0.02), and infrequent consumption of fresh vegetables (χ2=5.2 p=0.02) were associated with an increased prevalence of thinness. This study underscores the urgent need for comprehensive nutritional programs that could focus on vulnerable demographic groups. Interventions should focus on improving dietary intake.

## Background

Nutrition plays an important role in an individual’s physical and mental growth. The body’s demand for energy increases with age. Adequate nutrition from childhood to adulthood is critical for performance of physiological functions. Adolescence (11–19 years) is a critical age period in which an individual transitions from childhood to adulthood. The significant changes include growth, increases in height, and weight, hormonal changes, the appearance of secondary sexual characteristics, changes in voice, and cognitive development [1]. Nutritional availability and affordability greatly vary around the globe due to wealth accumulation by 1% of rich people and widening economic inequalities [2]. Most people who live in low- and middle-income countries face problems including poverty, illiteracy, inflation, low income, unemployment, and intra-generational nutritional issues such as stunting and thinness from childhood to adolescence. Stunting and thinness affect poverty-ridden people irrespective of gender. Adolescent females in the low and middle-income South Asian countries are no exception. United National International Children Emergency Fund (UNICEF) the prevalence of stunting and thinness among South Asian adolescent females was 11% and 39% respectively. South Asian adolescent females were consuming a poor diet that did not meet the dietary requirements for their physical growth. Stunting and thinness were more common in female adolescents from rural areas, large families, and unskilled, uneducated parents with low-income households [3]. Though South Asian females are more vulnerable to stunting and thinness adolescent males are also affected by stunting and thinness. The prevalence of stunting and thinness among teenage boys in Uttar Pradesh, India was 25.6% and 25.8% [4]. The prevalence of stunting and thinness among male adolescents in Chowhali, Bangladesh was 46.6% and 42.4% [5].

The population of Pakistan is 241 million with a growth rate of 2.55% [6]. The country’s gross domestic product (GDP) to debt ratio is 74.4% [7]. The World Health Organization has recommended that each country should spend at least 6% of the total GDP on the health of its people. However, last year the federal government of Pakistan allocated only PKR 24.25 billion for the health sector which is 2.8% of the total development budget and 0.05% of the GDP [8]. According to the Multidimensional Poverty Index (MPI) Survey in Pakistan, Balochistan has the highest level of multidimensional poverty, with 70% of its population considered poor. Khyber Pakhtunkhwa (KP) follows with 48%, and Sindh with 45%. Punjab has the lowest rate of multidimensional poverty at 30%, which is also below the national average of 39.1%. Over recent years, the levels of multidimensional poverty have generally decreased in most provinces except for Sindh. For example, Balochistan dropped slightly from 72.4% in 2014–15 to 70.5% in 2019–20. In KP, it decreased from 49.1% to 48.8%. In Punjab, it went down from 31.0% to 30.4%. However, in Sindh, multidimensional poverty increased from 43.1% in 2014–15 to 45.2% in 2019–20 [9]. Adolescents in such socioeconomic conditions face several challenges contributing tostunting and thinness. Such challenges include food insecurity, low income, large families, lack of education, and poor healthcare.

Previously conducted studies from various areas of Pakistan have focused on the prevalence of stunting and thinness among adolescents in either urban [10] or semi-urban areas, female adolescents [11], and late adolescents [12], Tharparkar is the deserted area with the highest rates of mortality in children under five years of age, previously conducted studies in Tharparkar have focused on stunting, thinness, and wasting in children under 5 years of age [13], school-aged children [14] micronutrient deficiency [15,16]. However, no study has identified the sociodemographic and dietary factors associated with stunting and thinness among adolescents in Tharparkar, Sindh, Pakistan. The objective of the study was to assess the sociodemographic and dietary factors associated with the prevalence of stunting and thinness among adolescents in Tharparkar.

## Methodology

### Study area

Tharparkar district is a deserted area by topography. Tharparkar has 7 Tahsils (sub-districts) including Mithi, Islamkot, Diplo, Kaloi, Chachro, Dahli, and Nangar Parkar. The vegetation, desert tourism, small businesses, and livestock are the main sources of livelihood. The region is affected by climate change. Poverty, hunger, low economic status, access to healthcare facilities and inadequate health facilities are common problems for people of Tharparkar. Poverty, Illiteracy, and lack of awareness of diet and its role in preventing thinness and stunting and their health consequences make the adolescent population vulnerable to nutrition inadequacy-related problems.

### Study setting

A survey-based cross-sectional study was conducted to assess the demographic and dietary risk factors associated with the prevalence of stunting and thinness among adolescents in Tharparkar District’s selected areas from June 2022 to August 2022. Data was collected using a multistage cluster sampling method. To ensure representation, the most populated villages from each sub-districts most populated Union councils were targeted. Within these selected villages, households were chosen randomly to participate in the survey. Subsequently, within the randomly selected households, adolescents were chosen for inclusion in the survey using a lottery method. This approach further added to the randomness of the selection process. The multistage cluster sampling method was designed to provide a comprehensive and representative sample of adolescents in Tharparkar for the survey.

### Sample size

The sample size was calculated using the following formula Sample Size (n) = [(Z^2) * p * (1 - p)]/ (E^2) Where: n: Sample size, - Z: Z-score corresponding to the desired confidence level approximately 1.96 for 95% confidence), p: Expected stunting prevalence (as a decimal) - E: Margin of Error (as a decimal). Because there is no previous literature on the prevalence of stunting and thinness among adolescents of Tharparkar. So, the average national prevalence of stunting and thinness of 25% percent was taken. Twenty-five percent expected prevalence (0.25), a 95% confidence level, and a 5% margin of error, as a result, a sample size of approximately 424 adolescents was needed for the study. We collected more than 600 samples. A total of 635 samples were collected. However, 36 samples were excluded for various reasons: age above 19 [4], age below 10 [4], married individuals [12], recent diarrhea cases [6], and outliers [10]. This left 599 samples for analysis. (Table 1).

**Table 1:** Sample flow chart.

Tharpakar District							
**Sub-Districtr**	**Nangar Parkar**	**Mithi**	**Chhachhro**	**Kaloi**	**Islamkot**	**Diplo**	**Dahli**
**Union Council**	**Nangar Parkar**	**Mithi**	**Chhachhro**	**Kaloi**	**Islamkot**	**Diplo**	**Dahli**
**Union Council**	**Satidera**	**Chelhar**	**Kantio**	**Bhitato**	**Sengahro**	**Jhirmiryo**	**Laplo**
**Union Council**	**Verawah**	**M.Veena**	**Mithrio Charan**	**Khetlari**	**Khario**	**Sobhiyar**	**Parno**
**Union Council**			**Rajoro**				
**Samples#**	**n=90**	**n=100**	**n=92**	**n=88**	**n=89**	**n=91**	**n=85**
		**Total Samples = 635**					
		**Samples Excluded**					
		**Age>19=4**					
		**Age<10=4**					
		**Married=12**					
		**Diarrhea last 15 days=6**					
		**Outliers=10**					
		**Samples included = 599**					

### Informed consent

Most of the participants were under 18 years old, after explaining the research project objectives to their parents, written informed consent for data collection was obtained from parents.

### Data collection

A team of trained data collectors was formed. The team was trained to collect data in a rural setting under the supervision of a trained Ph.D. student. All those participants whose permission was obtained were asked to go to station 1 for an interview about sociodemographic and dietary factors, then station 2 for anthropometry.

### Variable setting

The presence or absence of stunting and thinness were considered dependent variables. Independent variables included Demographic factors such as age, gender, and ethnicity, socioeconomic factors such as education level, parental education, siblings, income, and total family members, dietary factors such as breakfast, lunch, dinner, and type of diet, Height for age Z score (HAZ) above 0 was considered normal between -2 and -0.99 is considered mildly stunted, HAZ between -2 and -3 was considered Moderately stunted HAZ less than -3 was considered severely stunted. Body Mass Index for age Z score (BAZ) above 0 was considered normal between -2 and -0.99 is considered mild, BAZ between -2 and -3 was considered Moderately thin BAZ less than -3 was considered severely thin.

### Anthropometry

Participants were asked to remove heavy clothes, shoes, and other accessories. Weight and height were measured using calibrated scales placed on level surfaces. Weight and height were recorded to the nearest 0.1 kilograms and 0.5 inches, respectively. Height for age Z score (HAZ) and Body Mass Index for age Z score (BAZ) were calculated using the World Health Organization (WHO) recommended Anthro plus software.

#### Inclusion and exclusion criteria.

Healthy unmarried adolescents who permanently belong to district Tharparkar without any disease history in the past 3 months were included in the study. Unhealthy, married, under or over the age of adolescence with any disease history in the past 3 months were excluded from this study.

### Ethical approval

Ethical approval was obtained from the Research Ethical Review Committee of the University of Sindh, Jamshoro.

### Statistical analysis

Special Package for Social Sciences (SPSS) software version 23 was used to compute frequencies, mean, SD, and chi-square and binary logistic regression analysis. The significance was determined using a P value of 0.05. WHO Anthro Plus software was used to calculate the Z score.

## Results

### General characteristics of study participants

The overall mean age of the study participants was 14.11±2.43 years, and the mean weight was 36.842±8.83 kgs. The mean height of the study participants was 149.0151±11.27 centimeters. The mean height for age z score was -1.3094±1.17. The mean body mass index for age z score was -1.5473±1.27. The gender-wise mean values are shown in Table 2.

**Table 2:** General characteristics of Study participants.

General Characteristics	Overall (599)	Female (n=307)	Male (n=292)
Age in years	14.1134 ±2.43	14.3455±2.47	13.8693±2.37
Weight (kg)	36.842±8.83	36.820±7.23	36.866±10.26
Height (cm)	149.0151±11.27	147.6104±7.47	150.4920±14.07
HAZ	-1.3094±1.17	-1.3840±1.08	-1.2311±1.26
BAZ	-1.5473±1.27	-1.3360±1.16	-1.7696±1.35

### Basic demographic characteristics of the study participants

The demographic analysis revealed, 50% of the adolescents were of the age of early adolescence, 51.3% of the adolescents were females, 71.3% of the adolescents were literate, 56% of the adolescents had literate fathers and 86% of the adolescents had illiterate mothers, 87.4% of the adolescent’s fathers had income ≤33000 Pakistani Rupees (PKR), 62.5% of the adolescents had >7family members and 75% of the adolescents had ≤7 siblings. (Table 3).

**Table 3:** Basic demographic characteristics of study participants.

Characteristics.	Frequency	Percentage (%)
**Age**		
Early	299	49.9
Middle	190	31.7
Late	110	18.4
**Gender**		
Female	307	51.3
Male	292	48.7
**Area**		
Rural	490	81.8
Urban	109	18.2
**Education**		
Illiterate (0)	172	28.7
Primary (1–5)	137	22.9
Secondary (6–10)	248	41.4
Higher Secondary (>10)	42	7
**Father’s Education**		
Illiterate	260	43.4
Literate	339	56.6
**Mother’s Education**		
Illiterate	516	86.1
Literate	83	13.9
**Socioeconomic status**		
Monthly income ≤33000PKR	503	84.0
Monthly income ≥33100PKR	95	15.9
**Siblings**		
< 7	451	75.3
>7	148	24.7
**Family Members**		
< 7	227	37.9
>7	372	62.1

### Prevalence of stunting and its associated demographic and dietary factors

**Fig 1:**
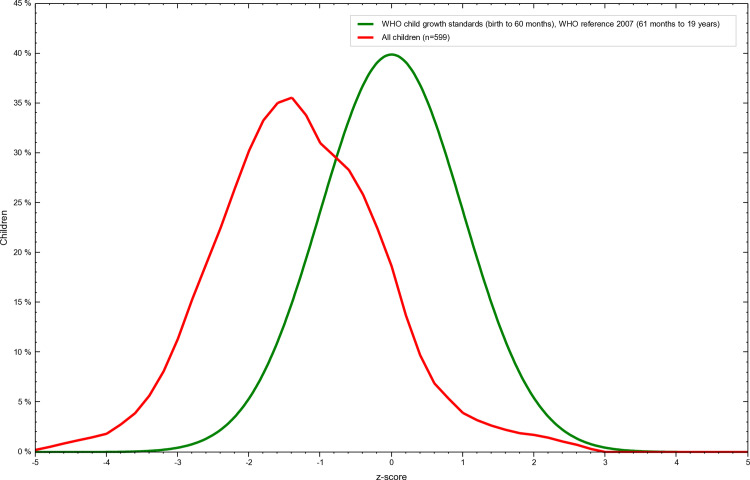
Overall Z-score distribution of stunting among adolescents of Tharparkar, Sindh, Pakistan. “This graph illustrates the overall Z-score distribution of height-for-age (stunting) among Adolescents of Tharparkar, Sindh, Pakistan generated using WHO AnthroPlus software. The Z-scores are calculated based on the 2007 WHO Growth Reference, with values below -2 standard deviations indicating stunting. The figure highlights the prevalence of malnutrition in the studied population and visually represents their growth and nutritional status relative to global standards.

**Fig 2:**
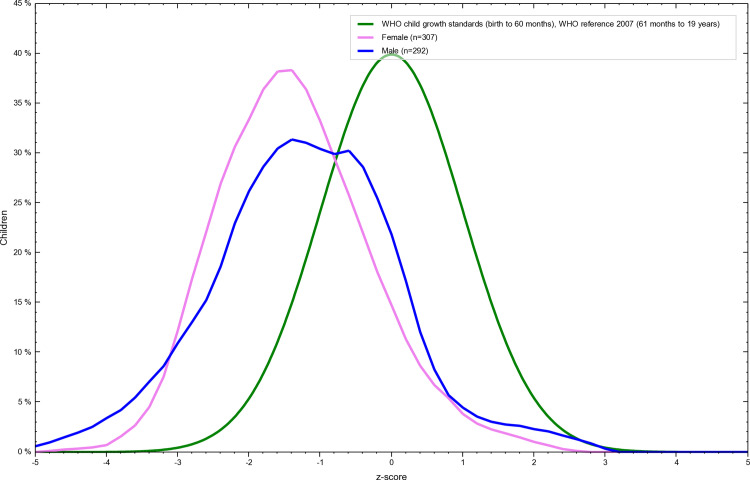
Gender wise Z-score distribution of stunting among adolescents of Tharparkar, Sindh, Pakistan. This graph illustrates the genderwise Z-score distribution of height-for-age (stunting) among Adolescents of Tharparkar, Sindh, Pakistan generated using WHO AnthroPlus software. The Z-scores are calculated based on the 2007 WHO Growth Reference, with values below -2 standard deviations indicating stunting. The figure highlights the prevalence of malnutrition in the studied population and visually represents their growth and nutritional status relative to global standards.“.

The overall prevalence of stunting was 27% among adolescents of Tharpakar. Twenty-eight percent of females and 26% of males were stunted. (Table 4 and Figs 1 and 2)

**Table 4:** Overall and gender-wise prevalence of stunting and its categories according to Z-score 2007 WHO reference.

Stunting	Frequency	Percentage
**Overall**		
Normal ≤0. Z-score	229	38.2%
Mild ≤1. Z-score	210	35.1%
Moderate ≤2. Z-score	125	20.9%
Severe ≤ 3. Z-score	35	5.8%
**Female**		
Normal ≤0. Z-score	103	33.6%
Mild ≤1. Z-score	117	38.1%
Moderate ≤2. Z-score	74	24.1%
Severe ≤ 3. Z-score	13	4.2%
**Male**		
Normal ≤0. Z-score	126	43.2%
Mild ≤1. Z-score	93	31.8%
Moderate ≤2. Z-score	51	17.5%
Severe ≤ 3. Z-score	22	7.5%

The late adolescence group was significantly associated with an increased prevalence of stunting (χ^2=^10.55 *p*=0.005). Illiterate and less educated groups (education ≤5 class) were associated with an increased prevalence of stunting (χ^2=^8.41 *p*=0.03). The rural area group was significantly associated with an increased prevalence of stunting (χ^2=^3.92 *p*=0.04) (Table 5). Dietary parameters are given in S1 Table. A binary logistic regression model examined the association between stunting and demographic factors. Primary and higher secondary education levels and middle adolescence stage are significantly associated with reduced odds of stunting (Table 6).

**Table 5:** Association of demographic factors with prevalence of stunting among adolescents of Tharparkar. Sindh, Pakistan.

demographic Factors	Stunting No	Stunting Yes	Chi-squire	P-value
**Age**				
Early	236 (78.9%)	63(21.1%)	10.558	.005
Middle	130 (68.4%)	60(31.6%)		
Late	72 (65.5%)	38(34.5%)		
**Gender**				
Female	220 (71.7%)	87(28.3%)	.684^a^	.408
Male	218 (74.7%)	74(25.3%)		
**Area**				
Rural	350 (71.4%)	140(28.6%)	3.928	.047
Urban	88 (80.7%)	21(19.3%)		
**Education**				
Illiterate (0)	119 (69.2%)	53 (30.8%)		
Primary (1–5)	109 (76.6%)	28 (20.4%)	8.41	0.03
Secondary (6–10)	174 (70.2%)	74 (29.8%)		
Higher Secondary (>10)	36 (85.7)	6 (14.3%)		
**Father’s Education**				
Illiterate	185 (71.2%)	75 (28.8%)	.905	.341
Literate	253 (74.6%)	86 (25.4%)		
**Mother’s Education**				
Illiterate	375 (72.7%)	141 (27.3%)	.379	.538
Literate	63 (75.9%)	20 (24.1%)		
**Socioeconomic status**				
Monthly income ≤33000PKR	329 (65.3%)	175 (34.7%)	.040	.841
Monthly income ≥33100PKR	61 (64.2%)	34 (35.8%)		
**Siblings**				
< 7	331 (73.4%)	120 (26.6%)	.068	.794
>7.	107 (72.3%)	41 (27.7%)		
**Family Members**				
< 7	171 (75.3%)	56 (24.7%)	.907	.341
>7	267 (71.8%)	105 (28.2%)		

**Table 6:** Binary logistic regression analysis stunting adjusted with demographic factors.

Variables in the Equation									
		B	S.E.	Wald	df	Sig.	Exp(B)	95% C.I.for EXP(B)	
								Lower	Upper
Step 1^a^	Gender (1)	-.096	.210	.210	1	.647	.908	.602	1.370
	Area (1)	.529	.280	3.578	1	.059	1.697	.981	2.936
	Illiterate			9.003	3	.029			
	Primary Education	1.177	.504	5.454	1	.020	3.244	1.208	8.712
	Secondary Education	.919	.524	3.079	1	.079	2.508	.898	7.003
	Higher Secondary Education	1.343	.493	7.430	1	.006	3.830	1.458	10.059
	Family Members (1)	-.148	.216	.468	1	.494	.862	.564	1.318
	Siblings (1)	.057	.238	.058	1	.809	1.059	.665	1.687
	Early Adolescence			13.793	2	.001			
	Middle Adolescence	-.951	.268	12.554	1	.000	.386	.228	.654
	Late Adolescence	-.426	.274	2.419	1	.120	.653	.382	1.117
	Constant	-1.925	.514	14.042	1	.000	.146		

^a^Variable(s) entered on step 1: Gender (1), Area (1), Education Level (Illiterate, Primary, Secondary, Higher Secondary), Family Members (1), Siblings (1), Adolescence Stage (Early, Middle, Late)

Outcome **variable**: Thinness status (0 = Not Stunted, 1 = Stunted)

Predictor **variables**: **Age**: Early, Middle and Late (Reference Category: Late), **Gender**: Male or Female (reference category = Male), **Socioeconomic status**: Categorical, coded as low, middle, high (reference category = High), **Education level**: Primary, Secondary, Higher (reference category = Higher), **Urban/Rural residence**: Place of residence (Urban = 1, Rural = 0; reference =Urban) Adjusted **odds ratio (OR)**: Odds ratio adjusted for age, gender, socioeconomic status, education level, and place of residence. 95**% Confidence Interval (CI)**: The range within which the true odds ratio is expected to fall, with 95% certainty.

### Prevalence of thinness and its associated demographic and dietary factors

**Fig 3:**
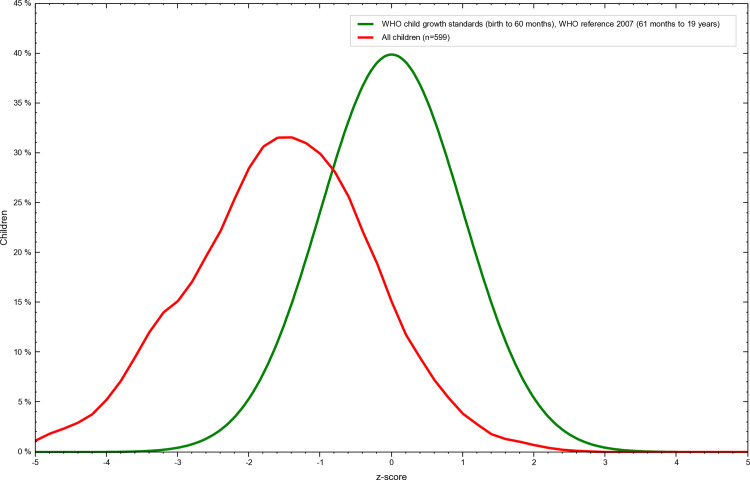
Overall Z-score distribution of thinness among adolescents of Tharparkar, Sindh, Pakistan. This graph illustrates the overall Z-score distribution of weight-for-age (thinness) among Adolescents of Tharparkar, Sindh, Pakistan generated using WHO AnthroPlus software. The Z-scores are calculated based on the 2007 WHO Growth Reference, with values below -2 standard deviations indicating stunting. The figure highlights the prevalence of malnutrition in the studied population and visually represents their growth and nutritional status relative to global standards.

**Fig 4:**
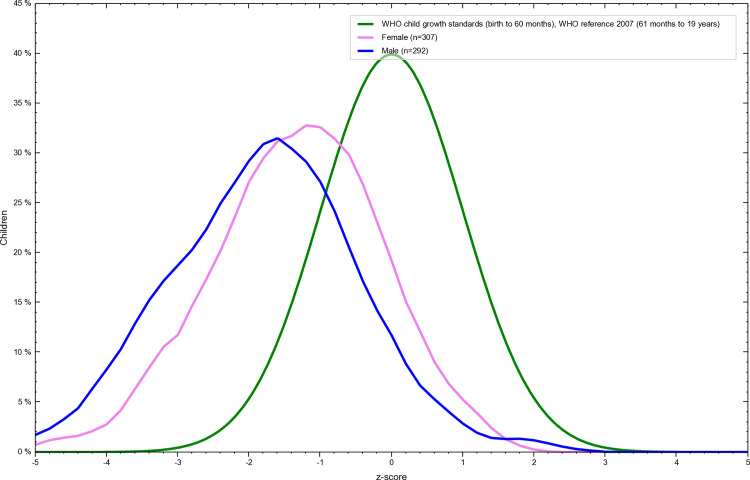
Gender wise Z-score distribution of thinness among adolescents of Tharparkar, Sindh, Pakistan. This graph illustrates the gender-wise Z-score distribution of weight-for-age (thinness) among Adolescents of Tharparkar, Sindh, Pakistan generated using WHO AnthroPlus software. The Z-scores are calculated based on the 2007 WHO Growth Reference, with values below -2 standard deviations indicating stunting. The figure highlights the prevalence of malnutrition in the studied population and visually represents their growth and nutritional status relative to global standards.

The overall prevalence of thinness was 35% among adolescents of Tharpakar. Twenty-eight percent of females and 42% of males were thin. (Table 7 and Figs 3 and 4).

**Table 7:** Overall and gender-wise prevalence of thinness and its categories according to Z-score 2007 WHO reference.

Thinness	Frequency	Percentage
**Overall**		
Normal ≤0. Z-score	198	33.1%
Mild ≤1. Z-score	192	32.1%
Moderate ≤2. Z-score	126	21.0%
Severe ≤ 3. Z-score	83	13.9%
**Female**		
Normal ≤0. Z-score	123	40%
Mild ≤1. Z-score	92	31%
Moderate ≤2. Z-score	56	18%
Severe ≤ 3. Z-score	30	10%
**Male**		
Normal ≤0. Z-score	75	25%
Mild ≤1. Z-score	94	32%
Moderate ≤2. Z-score	70	24%
Severe ≤ 3. Z-score	53	18%

The male group was significantly associated with an increased prevalence of thinness (χ^2=^13.11 *p*<0.0001). The literate group was associated with an increased prevalence of thinness (χ^2=^12.04 *p*=0.007). (Table 8) Infrequent consumption of eggs was associated with an increased prevalence of thinness (χ^2=^5.2 *p*=0.02). Infrequent consumption of fresh vegetables was associated with an increased prevalence of thinness (χ^2=^5.2 *p*=0.02). (Table 9). A binary logistic regression model examined the association between thinness and demographic factors. Gender (being male) and primary education are significant predictors of thinness. Males are at higher odds for thinness, while individuals with primary education have significantly lower odds of thinness compared to those who are illiterate (Table 10).

**Table 8:** Association of demographic factors with the prevalence of thinness among adolescents of Tharparkar, Sindh, Pakistan.

Sociodemographic Factors	Thinness No	Thinness Yes	Chi-squire	P-value
**Age**				
Early	200 (66.9%)	99 (33.1%)	2.210	.331
Middle	125 (65.8%)	65 (34.2%)		
Late	65 (59.1%)	45 (40.9%)		
**Gender**				
Female	221 (72.0%)	86 (28.0%)	13.116	.0001
Male	169 (57.9%)	123 (42.1%)		
**Area**				
Rural	317 (64.7%)	173 (35.3%)	.204	.652
Urban	73 (67.0%)	36 (33.0%)		
**Education**				
Illiterate (0)	125 (72.7%)	47 (27.3%)	12.04	.007
Primary (1–5)	97 (67.9%)	44 (32.1%)		
Secondary (6–10)	152 (61.3%)	97 (38.7%)		
Higher Secondary (>10)	20 (47.6%)	22 (52.4%)		
**Father’s Education**				
Illiterate	167 (64.2%)	93 (35.8%)	.156	.693
Literate	223 (65.8%)	116 (34.2%)		
**Mother’s Education**				
Illiterate	337 (65.3%)	179 (34.7%)	.067	.796
Literate	53 (63.9%)	30 (36.1%)		
**Socioeconomic status**				
Monthly income ≤33000PKR	329 (65.3%)	175 (34.7%)	.040	.841
Monthly income ≥33100PKR	61 (64.2%)	34 (35.8%)		
**Siblings**				
< 7	298 (66.1%)	153 (33.9%)	.751	.386
>7.	92 (62.2%)	56 (37.8%)		
**Family Members**				
< 7	155 (68.3%)	72 (31.7%)	1.620	.203
>7	235 (63.2%)	137 (36.8%)		

**Table 9:** Association of dietary factors with prevalence of thinness among adolescents of Tharparkar, Sindh, Pakistan.

Dietary Factors	Thinness No =(n) &%	Thinness Yes = (n) &%	Chi-squire	P-value
**Lassi**				
Infrequent	307 (65.0%)	165 (35.0%)	.004	.948
Frequent	83 (65.4%)	44 (34.6%)		
**Yogurt**				
Frequent	82 (63.1%)	48 (36.9%)	1.197	.550
Infrequent	69 (69.7%)	30 (30.3%)		
Non frequent	239 (64.6%)	131 (35.4%)		
**Milk**				
≤ 1 glass/day	323 (65.0%)	174 (35.0%)	.005	.945
≥ 2 glass/day	66 (65.3%)	35 (34.7%)		
**Butter**				
No	325 (65.9%)	168 (34.1%)	.813	.367
Yes	65 (61.3%)	41 (38.7%)		
**Chicken**				
No	308 (63.5%)	177 (36.5%)	2.884	.089
Yes	82 (71.9%)	32 (28.1%)		
**Egg**				
No	293 (62.7%)	174 (37.3%)	5.229	.022
Yes	97 (73.5%)	35 (26.5%)		
**Pulses**				
Frequent	185 (65.4%)	98 (34.6%)	.016	.898
Infrequent	205 (64.9%)	111 (35.1%)		
**Rice**				
Infrequent	201 (65.7%)	105 (34.3%)	.092	.762
Frequent	189 (64.5%)	104 (35.5%)		
**Fruit**				
No	133 (64.3%)	74 (35.7%)	.102	.749
Yes	257 (65.6%)	135 (34.4%)		
**Fresh Vegetables**	311 (67.6%) 79 (56.8%)	149 (32.4%) 60 (43.2%)	5.455	.020
Frequent				
Infrequent				
**Preserved Vegetables**				
Frequent	85 (59.9%)	57 (40.1%)	2.258	.133
Infrequent	305 (66.7%)	152 (33.3%)		

**Table 10:** Binary logistic regression analysis thinness adjusted with demographic factors.

Variables in the Equation									
		B	S.E.	Wald	df	Sig.	Exp(B)	95% C.I.for EXP(B)	
								Lower	Upper
Step 1^a^	Gender (1)	0.54	0.193	7.863	1	0.005	1.716	1.176	2.502
	Area (1)	0.241	0.242	0.989	1	0.32	1.272	0.792	2.045
	Illiterate			5.798	3	0.122			
	Primary Education	-0.83	0.395	4.427	1	0.035	0.436	0.201	0.945
	Secondary Education	-0.705	0.406	3.02	1	0.082	0.494	0.223	1.094
	Higher Secondary Education	-0.433	0.372	1.354	1	0.245	0.648	0.313	1.345
	Family Members (1)	-0.259	0.202	1.654	1	0.198	0.771	0.519	1.146
	Siblings (1)	-0.043	0.221	0.038	1	0.845	0.958	0.621	1.478
	Early Adolescence			1.464	2	0.481			
	Middle Adolescence	-0.277	0.26	1.132	1	0.287	0.758	0.455	1.263
	Late Adolescence	-0.307	0.267	1.319	1	0.251	0.736	0.436	1.242
	Constant	-0.164	0.388	0.179	1	0.672	0.849		

^a^Variable(s) entered on step 1: Gender (1), Area (1), Education Level (Illiterate, Primary, Secondary, Higher Secondary), Family Members (1), Siblings (1), Adolescence Stage (Early, Middle, Late)

Outcome **variable**: Thinness status (0 = Not Thin, 1 = Thin)

Predictor **variables**: **Age**: Early, Middle and Late (Reference Category: Late), **Gender**: Male or Female (reference category = Male), **Socioeconomic status**: Categorical, coded as low, middle, high (reference category = High), **Education level**: Primary, Secondary, Higher (reference category = Higher), **Urban/Rural residence**: Place of residence (Urban = 1, Rural = 0; reference =Urban). Adjusted **odds ratio (OR)**: Odds ratio adjusted for age, gender, socioeconomic status, education level, and place of residence. 95**% Confidence Interval (CI)**: The range within which the true odds ratio is expected to fall, with 95% certainty.

## Discussion

This study aimed to assess the prevalence and demographic factors associated with stunting and thinness among adolescents of Tharparkar Sindh. The overall prevalence of stunting and thinness was 27%, and 35% respectively. Key risk factors identified included age, gender, education, and dietary factors such as chicken, egg, and fresh vegetables. Our findings indicated that the overall prevalence of stunting falls within the range reported in previous research, which has documented rates from 22.72% [17] to 37% [10]. Regarding thinness, the prevalence of thinness slightly exceeds the range reported in previous research, which documented rates from 9.3 [12] to 31% [10].

In the context of gender differences, our results indicated that the prevalence of stunting is nearly similar between females 28%, and males 25% while the prevalence of thinness showed high variance with 42% in males and 28% in females, indicating potential disparity between the genders. This disparity has also been noted in other studies conducted in Nigeria, Ethiopia, and India [18–20]. This could be because of the increased energy demands of males which are not fulfilled compared to females and socio-economic and cultural factors could be attributed to explain this problem.

Demographic and dietary factors are important in explaining disparities in the prevalence of stunting and thinness. Our findings indicated that factors such as middle and late adolescent age group, rural area group, illiterate, and less educated group (participants with education up to class 5) were positively associated with increased prevalence of stunting among adolescents. Our findings are in line with previous studies [11,19].Our results showed that the male and educated groups were associated with an increased prevalence of thinness. Analysis of dietary factors showed that infrequent consumption of eggs and fresh vegetables was significantly associated with an increased prevalence of thinness although infrequent consumption of chicken did not show a statistically significant association, it showed a trend towards increased thinness. The findings of this study about the increased prevalence of thinness among males are consistent with other studies [18,19]. Several previous studies have shown that the illiterate group is a risk factor for the increased prevalence of thinness [21,22] Our findings suggest that an educated group is associated with an increased prevalence of thinness. Our finding could be due to the sample sizes in the last two educational classes (particularly the group with education more than the 10th class) being relatively small. The small sample size may lead to statistical variability. The study has limitations such as the small representation of the late adolescent age group, potential recall bias in dietary assessments, and causal inferences because of the cross-sectional study. These limitations must be considered in future studies.

The high prevalence of stunting and thinness among adolescents in our study underscores the urgent need for comprehensive nutritional programs that could focus on the demographic groups identified by this study. Interventions should focus on improving dietary diversity and addressing socioeconomic disparities.

In conclusion, this study highlights significant prevalence rates of stunting and thinness among adolescents and identifies critical risk factors. Addressing these issues through targeted public health interventions is essential for improving adolescent health and preventing long-term adverse outcomes. The Federal Government of Pakistan and the Provincial Government of Sindh should allocate resources to start nutrition programs in schools and communities for the adolescent population to prevent stunting and thinness among the adolescent population of Tharparkar.

## Supporting information

S1 Table Association of dietary factors with prevalence of stunting among adolescents of Tharparkar, Sindh, Pakistan. (DOCX)

S2 Table Data of Z-scores for height for age and weight for age of adolescents of Tharparkar, Sindh, Pakistan. NRPU 14406.
(XLSX)

## References

[1] Organization WH. Nutrition in adolescence: issues and challenges for the health sector: issues in adolescent health and development; 2005.

[2] Remington TFJSDI. Top-end wealth accumulation: the Forbes lists and wealth inequality. 2023;45(1).

[3] Fund UNICE. Adolescent and women’s nutrition: UNICEF is working in South Asia to improve the nutrition of adolescent girls and women; United Nation International Children Emergency ackn; 2024 [cited 2024 Jul 21]. Available from: https://www.unicef.org/rosa/what-we-do/nutrition/adolescent-and-womens-nutrition

[4] Kumar P, Srivastava S, Chauhan S, Patel R, Marbaniang SP, Dhillon P. Associated factors and socio-economic inequality in the prevalence of thinness and stunting among adolescent boys and girls in Uttar Pradesh and Bihar, India. PLoS One. 2021;16(2):e0247526. doi: 10.1371/journal.pone.0247526 33626097 PMC7904204

[5] Rahman M, Karim R. Prevalence of stunting and thinness among adolescents in rural area of Bangladesh. J Agric Sci Res. 2014;4(1):39.

[6] Announcement of results of 7th population and housing census-2023 ‘The Digital Census’ [Internet]. Pakistan Bureau of Statistics; 2023 [cited 2024 Jul 19]. Available from: https://www.pbs.gov.pk/sites/default/files/population/2023/Press%20Release.pdf

[7] Pakistan’s debt and liabilities-summary [Internet]; 2024. Available from: https://www.sbp.org.pk/ecodata/Summary.pdf

[8] Saeed J. Analyzing health budget 2023 in context of wellbeing of people: sustainable development policy institute; 2023 [cited 2024 Jul 19]. Available from: https://sdpi.org/analyzing-health-budget-2023-in-context-of-wellbeing-of-people/news_detail#:~:text=In%20Pakistan%2C%20the%20provincial%20and,0.05%20per%20cent%20of%20GDP

[9] Nasir Iqbal HA. Multidimensional poverty in Pakistan report Pakistan Institute of Development Economics; 2024 Jul 21. Report No.

[10] Campisi SC, Humayun KN, Rizvi A, Lou W, Söder O, Bhutta ZA. Later puberty onset among chronically undernourished adolescents living in a Karachi Slum, Pakistan. Acta Paediatr. 2020;109(5):1019–25. doi: 10.1111/apa.15053 31606907

[11] Samo A, Laghari Z, Memon A, Baig N, Memon S, Shaikh S. Prevalence and sociodemographic factors associated with stunting and thinness in adolescent females: a cross sectional study from Pakistan. J Pak Med Assoc. 2022;72(3):471–6. 35320227 10.47391/JPMA.1757

[12] Baxter J-A, Wasan Y, Hussain A, Soofi S, Ahmed I, Bhutta Z. Drivers of malnutrition among late adolescent and young women in rural Pakistan: a cross-sectional assessment of the MaPPS trial. BMJ Open. 2023;13(5):e063734. 10.1136/bmjopen-2022-063734 PMC10230942 37221027

[13] Menghwar B, Laghari ZA, Memon SF, Warsi J, Shaikh SA, Baig NM. Prevalence of malnutrition in children under five years’ age in District Tharparkar Sindh, Pakistan. J Pak Med Assoc. 2022;72(1):33–6. doi: 10.47391/JPMA.20-540 35099434

[14] Kapoor A, Channa N, Soomro A, Tunio S, Khand T, Memon N. Malnutrition and clinical manifestations in school going children at district Tharparkar, Sindh, Pakistan. RMJ. 2018;43(1):115–9.

[15] Kapoor A, Fatima F, Ahsan N, Shaikh GS, Solangi DA, Shanker B. Vitamin D deficiency among well nourished and malnourished children of school going age at District Tharparkar. PJMHS. 2022;16(7):873–5. doi: 10.53350/pjmhs22167873

[16] Kapoor A, Baig F, Channa NA, Othman SS, Abualhamael SA, Baig M. Estimation of calories intake, iron, zinc, and selenium among children of the underprivileged area in Sindh, Pakistan. PLoS One. 2024;19(6):e0304277. doi: 10.1371/journal.pone.0304277 38917170 PMC11198758

[17] Qaisar R, Karim A. A comparison of international and national references to measure the prevalence of stunting in Pakistani school-age girls. Sci Rep. 2022;12(1):5501. doi: 10.1038/s41598-022-09511-3 35365745 PMC8976039

[18] Mijinyawa M, Yusuf S, Gezawa I, Musa B, Uloko A. Prevalence of thinness among adolescents in Kano, Northwestern Nigeria. Nig J Basic Appl Sci. 2014;11(1):24–9.

[19] Mengesha DK, Prasad RPCJ, Asres DT. Prevalence and associated factors of thinness among adolescent students in Finote Selam Town, Northwest Ethiopia. ScientificWorldJournal. 2020;2020:9170301. doi: 10.1155/2020/9170301 32565753 PMC7288196

[20] Mondal N, Sen J. Prevalence of stunting and thinness among rural adolescents of Darjeeling district, West Bengal, India. J Indian Public Health. 2010;7(1).

[21] Kumar P, Srivastava S, Chauhan S, Patel R, Marbaniang SP, Dhillon P. Associated factors and socio-economic inequality in the prevalence of thinness and stunting among adolescent boys and girls in Uttar Pradesh and Bihar, India. PLoS One. 2021;16(2):e0247526. doi: 10.1371/journal.pone.0247526 33626097 PMC7904204

[22] Gebregyorgis T, Tadesse T, Atenafu A. Prevalence of thinness and stunting and associated factors among adolescent school girls in Adwa Town, North Ethiopia. Int J Food Sci. 2016;2016:8323982. doi: 10.1155/2016/8323982 27294107 PMC4884871

