# Supplementary material for: Factors and determinants associated with prevalence of stunting and thinness among adolescents of Tharparkar, Sindh, Pakistan: A community-based study
Source: PLoS One. 2025 Apr 23;20(4):e0318055. doi: 10.1371/journal.pone.0318055 (PMC12017537; doi:10.1371/journal.pone.0318055)
Supplement: S1 Table — (DOCX) [file pone.0318055.s001.docx]

**Table S1. Association of dietary factors with prevalence of stunting among adolescents of Tharparkar, Sindh, Pakistan.**

| **Dietary Factors** | **Stunting No**  **=(n) &%** | **Stunting Yes**  **= (n) &%** | **Chi-squire** | **P-value** |
| --- | --- | --- | --- | --- |
| **Lassi**  Infrequent  Frequent | 341 (72.2%)  97 (76.4%) | 131 (27.8%)  30 (23.6%) | .869 | .351 |
| **Yogurt**  Frequent  Infrequent  Non frequent | 101 (77.7%)  71 (71.7%)  266 (71.9%) | 29 (22.3%)  28 (28.3%)  104 (28.1%) | 1.766 | .414 |
| **Milk**  ≤ 1 glass/day  ≥ 2 glass/day | 360 (72.4%)  77 (76.2%) | 137 (27.6%)  24 (23.8%) | .617 | .432 |
| **Butter**  No  Yes | 360 (73.0%)  78 (73.6%) | 133 (27.0%)  28 (26.4%) | .014 | .906 |
| **Chicken**  NO  Yes | 351 (72.4%)  87 (76.3%) | 134 (27.6%)  27 (23.7%) | .731 | .393 |
| **Egg**  No  Yes | 344 (73.7%)  94 (71.2%) | 123 (26.3%)  38 (28.8%) | .314 | .575 |
| **Pulses**  Frequent  Infrequent | 210 (74.2%)  228 (72.2%) | 73 (25.8%)  88 (27.8%) | .320 | .571 |
| **Rice**  Infrequent  Frequent | 218 (71.2%)  220 (75.1%) | 88 (28.8%)  73 (24.9%) | 1.125 | .289 |
| **Fruit**  No  Yes | 155 (74.9%)  283 (72.2%) | 52 (25.1%)  109 (27.8%) | .497 | .481 |
| **Fresh Vegetables** Frequent  Infrequent | 337 (73.3%)  101 (72.7%) | 123 (26.7%)  38 (27.3%) | .019 | .889 |
| **Preserved Vegetables**  Frequent  Infrequent | 99 (69.7%)  339 (74.2%) | 43 (30.3%)  118 (25.8%) | 1.097 | .295 |
