# Supplementary material for: Factors and determinants associated with prevalence of stunting and thinness among adolescents of Tharparkar, Sindh, Pakistan: A community-based study
Source: PLoS One. 2025 Apr 23;20(4):e0318055. doi: 10.1371/journal.pone.0318055 (PMC12017537; doi:10.1371/journal.pone.0318055)
Supplement: Attachment — Submitted filename: Response to Reviewers.docx [file pone.0318055.s004.docx]

Dear Dr. Turk,

Thank you for the opportunity to revise our manuscript and for the valuable feedback provided by the editorial team and reviewers. We appreciate the thoughtful consideration given to our work and are pleased to hear that our study has been recognized for its merit.

We have carefully reviewed each of the comments raised during the review process. In response, we have made the following changes to enhance the manuscript and address the specific points raised:

Journal Requirements:

1. When submitting your revision, we need you to address these additional requirements.

   Please ensure that your manuscript meets PLOS ONE's style requirements, including those for file naming. The PLOS ONE style templates can be found at
   <https://journals.plos.org/plosone/s/file?id=wjVg/PLOSOne_formatting_sample_main_body.pdf> and
   <https://journals.plos.org/plosone/s/file?id=ba62/PLOSOne_formatting_sample_title_authors_affiliations.pdf>

**Answer#** Thank you for your comment. We have updated the reference style as indicated.

2. Thank you for stating the following financial disclosure: "This study was funded by the research grant received from the Higher Education Commission, Islamabad through the National Research Program for Universities (NRPU) project number 14406 for 2021-2024."Please state what role the funders took in the study. If the funders had no role, please state: "The funders had no role in study design, data collection and analysis, decision to publish, or manuscript preparation. “If this statement is not correct you must amend it as needed. Please include this amended Role of Funder statement in your cover letter; we will change the online submission form on your behalf.

**Answer#** Thank you for your comment. We have modified the statement as suggested and included this information in manuscript revised file.

3. In the online submission form, you indicated that [Data can be provided by authors only on reasonable request to Principal Investigator on [ayazsamo@usindh.edu.pk](mailto:ayazsamo@usindh.edu.pk)].

All PLOS journals now require all data underlying the findings described in their manuscript to be freely available to other researchers, either 1. In a public repository, 2. Within the manuscript itself, or 3. Uploaded as supplementary information.
This policy applies to all data except where public deposition would breach compliance with the protocol approved by your research ethics board. If your data cannot be made publicly available for ethical or legal reasons (e.g., public availability would compromise patient privacy), please explain your reasons on resubmission and your exemption request will be escalated for approval.

4. When completing the data availability statement of the submission form, you indicated that you will make your data available on acceptance. We strongly recommend all authors decide on a data sharing plan before acceptance, as the process can be lengthy and hold up publication timelines. Please note that, though access restrictions are acceptable now, your entire data will need to be made freely accessible if your manuscript is accepted for publication. This policy applies to all data except where public deposition would breach compliance with the protocol approved by your research ethics board. If you are unable to adhere to our open data policy, please kindly revise your statement to explain your reasoning and we will seek the editor's input on an exemption. Please be assured that, once you have provided your new statement, the assessment of your exemption will not hold up the peer review process.

**Answer of point number 3 and 4#** Thank you for your guidance on the data availability policy. We understand and support the journal’s commitment to transparency and accessibility. However, due to its nature, we have some limitations regarding the unrestricted sharing of our data.

Our dataset contains demographic information that could potentially allow for the identification of participants if shared publicly. Additionally, as this is our primary data, we would prefer to share it only with researchers or research groups who agree to acknowledge our contribution by including our team in their subsequent publications. This ensures proper recognition for the extensive work involved in gathering this data while also safeguarding participant privacy.

Therefore, we request an exemption from the open data policy to allow for data sharing under these conditions. We are fully prepared to make the data available upon request to the editorial team for review purposes and to researchers who agree to our stipulations on acknowledgment. We trust this approach aligns with the journal’s standards for accessibility while addressing our concerns about data ownership and participant confidentiality.

5. Your ethics statement should only appear in the Methods section of your manuscript. If your ethics statement is written in any section besides the Methods, please move it to the Methods section and delete it from any other section. Please ensure that your ethics statement is included in your manuscript, as the ethics statement entered into the online submission form will not be published alongside your manuscript.

**Answer:** The ethical statement is provided in methodology section.

6. We note that there is identifying data in the Supporting Information file <Table S1.xlsx>. Due to the inclusion of these potentially identifying data, we have removed this file from your file inventory. Prior to sharing human research participant data, authors should consult with an ethics committee to ensure data are shared in accordance with participant consent and all applicable local laws.

Data sharing should never compromise participant privacy. It is therefore not appropriate to publicly share personally identifiable data on human research participants. The following are examples of data that should not be shared:

-Name, initials, physical address
-Ages more specific than whole numbers
-Internet protocol (IP) address
-Specific dates (birth dates, death dates, examination dates, etc.)
-Contact information such as phone number or email address
-Location data
-ID numbers that seem specific (long numbers, include initials, titled “Hospital ID”) rather than random (small numbers in numerical order)

Data that are not directly identifying may also be inappropriate to share, as in combination they can become identifying. For example, data collected from a small group of participants, vulnerable populations, or private groups should not be shared if they involve indirect identifiers (such as sex, ethnicity, location, etc.) that may risk the identification of study participants.

Additional guidance on preparing raw data for publication can be found in our Data Policy (<https://journals.plos.org/plosone/s/data-availability#loc-human-research-participant-data-and-other-sensitive-data>) and in the following article: <http://www.bmj.com/content/340/bmj.c181.long>.

Please remove or anonymize all personal information, ensure that the data shared are in accordance with participant consent, and re-upload a fully anonymized data set. Please note that spreadsheet columns with personal information must be removed and not hidden as all hidden columns will appear in the published file.

7. Please include captions for your Supporting Information files at the end of your manuscript, and update any in-text citations to match accordingly. Please see our Supporting Information guidelines for more information: <http://journals.plos.org/plosone/s/supporting-information>.

Additional Editor Comments:

Currently we have had one reviewer provide feedback on your manuscript with comments as follows:

[Note: HTML markup is below. Please do not edit.]

Reviewers' comments:

Reviewer's Responses to Questions

**Comments to the Author**

1. Is the manuscript technically sound, and do the data support the conclusions?

The manuscript must describe a technically sound piece of scientific research with data that supports the conclusions. Experiments must have been conducted rigorously, with appropriate controls, replication, and sample sizes. The conclusions must be drawn appropriately based on the data presented.

**Reviewer #1: Yes**

**Answer#** Thankful to reviewer for feedback.

2. Has the statistical analysis been performed appropriately and rigorously?

Reviewer #1: No

**Answer#** Thankful to reviewer for feedback. We have added additional statistical analysis as suggested by reviewers.

3. Have the authors made all data underlying the findings in their manuscript fully available?

The [PLOS Data policy](http://track.editorialmanager.com/CL0/http:%2F%2Fwww.plosone.org%2Fstatic%2Fpolicies.action%23sharing/1/010f0192e8a947b5-ad255a9d-09fb-4963-b615-4a605b9f7949-000000/X05mwkZUluBAycnAMR98IksBiw0SXZRh7ZYbJwHvybc=183) requires authors to make all data underlying the findings described in their manuscript fully available without restriction, with rare exception (please refer to the Data Availability Statement in the manuscript PDF file). The data should be provided as part of the manuscript or its supporting information, or deposited to a public repository. For example, in addition to summary statistics, the data points behind means, medians and variance measures should be available. If there are restrictions on publicly sharing data—e.g. participant privacy or use of data from a third party—those must be specified.

Reviewer #1: Yes

**Answer#** Thankful to reviewer for feedback.

4. Is the manuscript presented in an intelligible fashion and written in standard English?

PLOS ONE does not copyedit accepted manuscripts, so the language in submitted articles must be clear, correct, and unambiguous. Any typographical or grammatical errors should be corrected at revision, so please note any specific errors here.

Reviewer #1: Yes

**Answer#** Thankful to reviewer for feedback.

5. Review Comments to the Author

Please use the space provided to explain your answers to the questions above. You may also include additional comments for the author, including concerns about dual publication, research ethics, or publication ethics. (Please upload your review as an attachment if it exceeds 20,000 characters)

Reviewer #1: In this study, the authors examine the factors and determinants linked to the prevalence of stunting and thinness among adolescents in Tharparkar, Sindh, Pakistan. The overall study design and methodology are appropriate for this journal. The authors also report significant associations of stunting and thinness with various risk factors.
**Major comments:**
1. Were any of the models adjusted for confounding factors? It would be interesting to see the results in the presence of different confounding factors.

**Answer#** We are very thankful to the reviewer for this comment. We have adjusted the stunting and thinness based on the demographic factors we have studied. A binary logistic regression model examined the association between stunting and demographic factors. Primary and higher secondary education levels and middle adolescence stage are significantly associated with reduced odds of stunting (Table 6). Males are at higher odds for thinness, while individuals with primary education have significantly lower odds of thinness compared to those who are illiterate (Table 10).
2. The criteria for determining education levels are unclear. How was parental literacy assessed? It would be beneficial to explore the association between educational status (years of education) and stunting/thinness.

**Answer#** We are grateful to the reviewer for this comment. Education was determined by 0 means illiterate, 1-5 means primary education, 6-10 means secondary education, and >10 means higher secondary education. We have explored the association between educational status and stunting and thinness. Please follow Chi-square and logistic regression analysis tables.
**Minor comments:**
1. What do "sibling 1" and "sibling 2" in Table 3 refer to?

**Answer#** We are very thankful to the reviewer for pointing out this mistake. Siblings have been categorized into 2 groups sibling 1 indicates the number of siblings <7 and Sibling 2 indicates the number of siblings >7
2. The manuscript requires thorough English editing.

**Answer#** We have modified the language where needed.
